# Structural Optimization and Trap Effects on the Output Performance of 4H-SiC Betavoltaic Cell

**DOI:** 10.3390/nano15211625

**Published:** 2025-10-24

**Authors:** Kyeong Min Kim, In Man Kang, Jae Hwa Seo, Young Jun Yoon, Kibeom Kim

**Affiliations:** 1 Department of Electronics Engineering, Gyeongkuk National University, Andong 36729, Republic of Korea; 20255007@student.anu.ac.kr; 2 School of Electronic and Electrical Engineering, Kyungpook National University, Daegu 41566, Republic of Korea; imkang@ee.knu.ac.kr; 3 Advanced Semiconductor Research Center, Korea Electrotechnology Research Institute, Changwon 51543, Republic of Korea; 4 School of Electronic & Mechanical Engineering, Gyeongkuk National University, Andong 36729, Republic of Korea; 5 Department of Electronic Engineering, Soonchunhyang University, Asan 31538, Republic of Korea

**Keywords:** betavoltaic cell, 4H-Silicon Carbide (4H-SiC), TCAD simulation, trap, output power density

## Abstract

In this study, structural optimization and trap effect analysis of a 4H-SiC–based p–i–n betavoltaic (BV) cell were performed using Silvaco ATLAS TCAD (version 5.30.0.R) simulations combined with an electron-beam (e-beam) irradiation model. First, the optimum device structure was derived by varying the thickness of the intrinsic layer (i-layer), the thickness of the p-layer, and the doping concentration of the i-layer. Under 17 keV e-beam irradiation, the electron–hole pairs generated in the i-layer were effectively separated and transported by the internal electric field, thereby contributing to the short-circuit current density (J_SC_), open-circuit voltage (V_OC_), and maximum output power density (P_out_max_). Subsequently, to investigate the effects of traps, donor- and acceptor-like traps were introduced either individually or simultaneously, and their densities were varied to evaluate the changes in device performance. The simulation results revealed that traps degraded the performance through charge capture and recombination, with acceptor-like traps exhibiting the most pronounced impact. In particular, acceptor-like traps in the i-layer significantly reduced V_OC_ from 2.47 V to 2.07 V and P_out_max_ from 3.08 μW/cm^2^ to 2.28 μW/cm^2^, demonstrating that the i-layer is the most sensitive region to performance degradation. These findings indicate that effective control of trap states within the i-layer is a critical factor for realizing high-efficiency and high-reliability SiC-based betavoltaic cells.

## 1. Introduction

Betavoltaic (BV) cells are devices that convert the kinetic energy of beta(*β*)-particles emitted from radioactive isotopes into electrical energy. They have attracted significant attention as next-generation power sources for microelectronic devices, implantable biomedical systems, and power supplies for space and extreme environments, where long-term stable power delivery is required [1,2,3,4,5]. In particular, compared to conventional chemical microbatteries, BV cells offer a long operational lifetime by utilizing isotopes with half-lives of several decades, while ensuring stable operation even under harsh conditions such as high temperature and intense radiation [6].

The performance of BV cells is strongly correlated with the physical properties of the employed semiconductor materials. Various semiconductor-based BV cells have been investigated, including those utilizing Si [7,8], GaAs [9], GaN [10,11,12], and SiC [13,14]. Each material presents unique advantages and limitations. However, SiC stands out as a promising candidate due to its wide bandgap of ~3.26 eV, high thermal conductivity, mechanical and thermal stability, and relatively small performance degradation under radiation exposure [15,16,17]. To provide a clearer comparison, the representative material properties of various semiconductor candidates for betavoltaic cells are summarized in Table 1 [18,19,20,21,22]. Among the various SiC polytypes, 4H-SiC demonstrates outstanding resistance to lattice damage induced by energy particle irradiation and partial defect recovery capability, making it highly suitable for long-term reliable operation [23,24].

Previous studies have primarily focused on structural optimization [25,26], while systematic investigations into the influence of trap states on the electrical performance of SiC-based BV cells remain limited. In practice, however, defects or traps inevitably form during device fabrication and radiation exposure [27,28]. These traps act as recombination centers for electron–hole pairs, reducing the short-circuit current density (J_SC_) and open-circuit voltage (V_OC_) and ultimately degrading the output power characteristics. Therefore, to fully understand the device performance, it is necessary to consider not only structural optimization but also trap effects.

In this work, we investigate 4H-SiC–based p–i–n BV cells. First, structural optimization was conducted by varying the intrinsic layer (i-layer) thickness, p-layer thickness, and i-layer doping concentration. Subsequently, the impacts of donor- and acceptor-like traps were systematically analyzed with respect to their type, density, and layer-specific location (p-, i-, or n-layer) using TCAD simulations. In addition, an electron-beam (e-beam) irradiation model was introduced to evaluate the effects of traps on J_SC_, V_OC_, and the maximum output power density (P_out_max_).

## 2. Device Structure and Simulation Method

**Figure 1:**
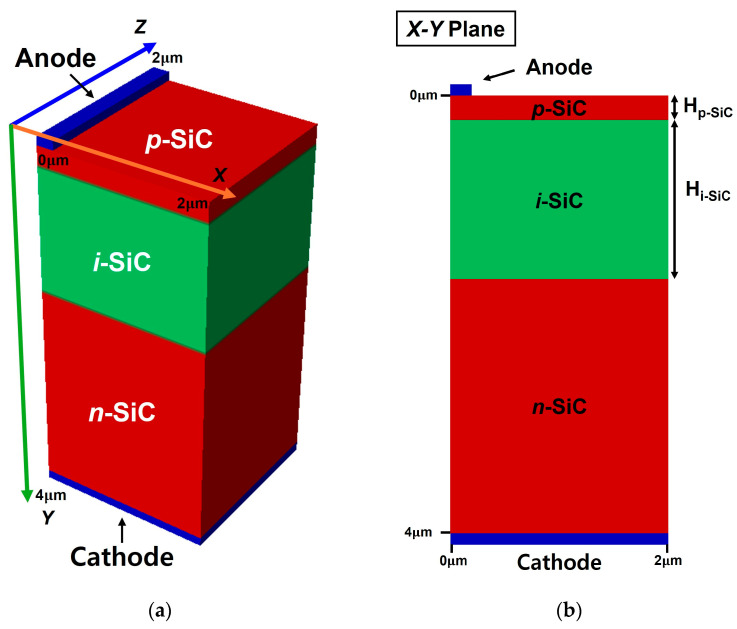
(**a**) Three-dimensional schematic of the simulated 4H-SiC p–i–n BV cell structure and (**b**) its cross-sectional view (*x–y* plane).

In this study, three-dimensional (3D) simulations were performed for a 4H-SiC–based p–i–n BV cell using the Silvaco ATLAS TCAD (version 5.30.0.R) simulator [29]. Figure 1a shows the schematic of the device, while Figure 1b illustrates its *x–z* cross-sectional view. The active region was defined with a cross-sectional area of 2 μm × 2 μm, and the total current obtained from the 3D model was normalized by this area (4 × 10^−8^ cm^2^) to calculate the current density (J). The overall device dimensions were set to 2 μm × 4 μm × 4 μm. This size was chosen as a balanced configuration to ensure sufficient mesh density while avoiding excessive computational cost. The top and bottom contacts were configured as the cathode and anode electrodes, respectively, forming a typical p–i–n diode structure consisting of p-, i-, and n-layers.

The simulated structure reflects a realistic fabrication process: an n^+^ 4H-SiC substrate was used, on which an n^−^ quasi-intrinsic layer was epitaxially grown. A p-type region was subsequently formed through ion implantation, resulting in a p–i–n structure, followed by electrode processing to realize a manufacturable device.

Carrier transport was modeled using the drift–diffusion equation, while recombination and tunneling effects were incorporated via Shockley–Read–Hall (SRH) recombination, trap-assisted tunneling (TAT), and Auger recombination models. Carrier mobility and saturation velocity were assigned based on 4H-SiC material parameters [30].

**Figure 2:**
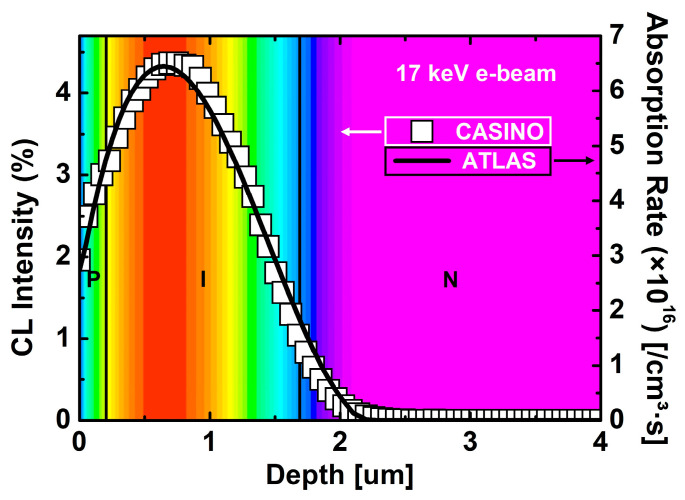
Depth-dependent absorption rate and CL intensity profiles under 17 keV e-beam irradiation, obtained from CASINO (Monte Carlo simulation) and ATLAS (TCAD simulation).

The e-beam irradiation condition was set at 17 keV, corresponding to the average beta-particle energy of Ni-63 (≈60 keV maximum, 17 keV mean energy), representing the typical spectrum-weighted energy deposition in Ni-63 sources [31]. The depth-dependent generation rate of electron–hole pairs (EHPs) was extracted from CASINO Monte Carlo simulations and implemented in ATLAS [32,33]. As shown in Figure 2, 17 keV electrons penetrated to a depth of ~2.3 μm, with a peak absorption rate at approximately 0.75 μm.

The majority of EHPs generated in this region were effectively separated and transported by the internal electric field of the i-layer in the p–i–n structure, directly contributing to J_SC_ and V_OC_. Therefore, the i-layer thickness (H_i-SiC_) and doping concentration (D_i-SiC_) are critical design parameters determining the maximum output power density (P_out_max_).

In this study, the e-beam irradiation model assumes instantaneous electron–hole pair generation at a fixed energy of 17 keV. Although cumulative defect evolution was not directly modeled, the degradation trend with increasing trap density reflects the expected influence of dose accumulation. Detailed dose-dependent defect dynamics will be further investigated in future work.

For the analysis of trap effects, it was first necessary to establish a baseline optimized structure. Structural optimization was performed by varying the i-layer thickness, the p-layer thickness, and the i-layer doping concentration as the main parameters. The i-layer thickness was varied from 0.9 to 2.6 μm, while the p-layer thickness was adjusted within 0.2–0.4 μm. The doping concentrations of the p- and n-layers were fixed at 1 × 10^20^ cm^−3^, while the i-layer doping was set to n-type 1 × 10^15^, 1 × 10^16^, and 5 × 10^16^ cm^−3^ for comparison. Electrical characteristics were evaluated using current–voltage (J–V) curves, with J_SC_, V_OC_, and P_out_max_ defined as the performance metrics.

Subsequently, trap effect analysis was conducted based on the determined optimized structure. Both donor- and acceptor-like traps were considered. Their energy levels were set to donor-like trap = E_V_ + 1.6 eV and acceptor-like trap = E_C_ − 0.63 eV, reflecting the deep-level trap states commonly reported in 4H-SiC [34]. Additionally, the capture cross-sections and degeneracy factors used in this study were σ_n_ = 2 × 10^−14^ cm^2^ and σ_p_ = 3.5 × 10^−14^ cm^2^ for the acceptor-like trap, and σ_n_ = σ_p_ = 1 × 10^−15^ cm^2^ for the donor-like trap, with a degeneracy factor of 1 for both traps [28,34]. The trap density was varied from 1 × 10^14^ to 5 × 10^15^ cm^−3^. Four trap conditions were analyzed: without traps, donor-like traps only, acceptor-like traps only, and both donor- and acceptor-like traps simultaneously. Furthermore, for the more influential trap type, its spatial distribution was analyzed by selectively placing it in the p-, i-, and n-layers to evaluate layer-dependent performance variations.

## 3. Results and Discussion

**Figure 3:**
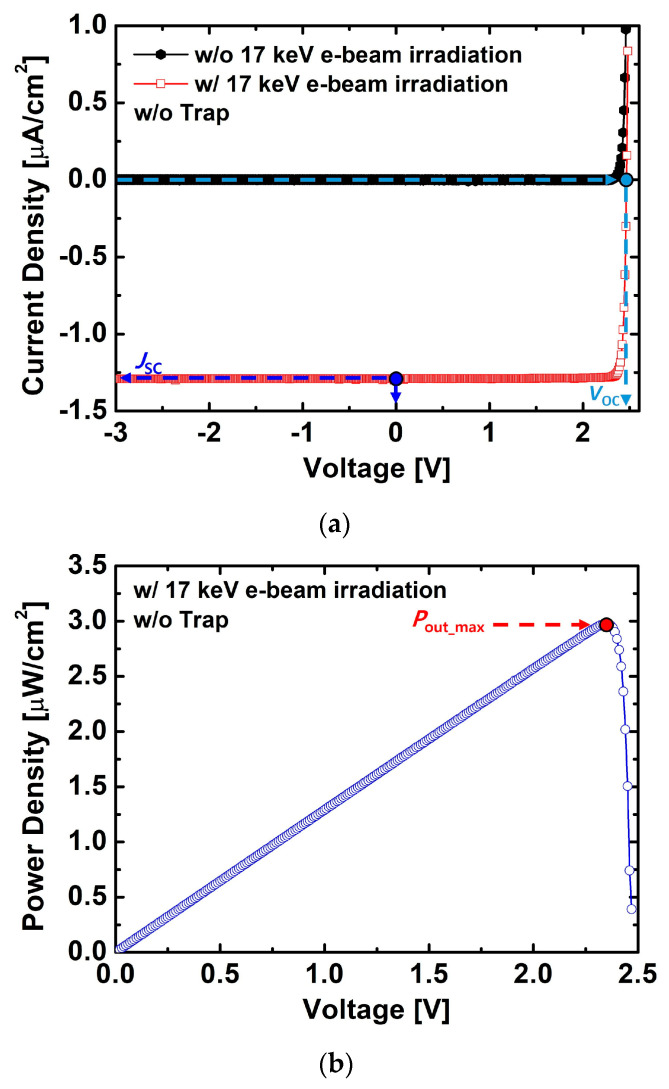
(**a**) J–V characteristics before and after 17 keV e-beam irradiation. (**b**) P–V characteristics under 17 keV e-beam irradiation, indicating P_out_max_. The structure parameters were H_p-SiC_ = 0.2 μm, H_i-SiC_ = 1.5 μm, D_p-SiC_ = 1 × 10^20^ cm^−3^, D_i-SiC_ = 1 × 10^15^ cm^−3^, and D_n-SiC_ = 1 × 10^20^ cm^−3^.

Figure 3a compares the current–voltage (J–V) characteristics of the device before and after 17 keV e-beam irradiation. After irradiation, the reverse current density of the device clearly increased. This is attributed to the generation of electron–hole pairs (EHPs) by the incident high-energy electrons, which were separated and transported by the internal electric field, thereby contributing to the current. From this structure, the extracted J_SC_ and V_OC_ were −1.29 μA/cm^2^ and 2.466 V, respectively. Here, J_SC_ and V_OC_ represent the current density at 0 V and the voltage at 0 A/cm^2^, respectively. The p-SiC (anode) and n-SiC (cathode) contacts were assumed to be ideal Ohmic contacts with identical resistances, as both regions were equally doped. The contact resistance was not included to focus on the relative impact of structural and trap-related parameters.

The power–voltage (P–V) characteristics obtained under e-beam irradiation are shown in Figure 3b. The output power density gradually increased with increasing voltage, reaching a maximum at approximately 2.35 V, where P_out_max_ was 2.98 μW/cm^2^. This result can be interpreted as the effective collection and conversion of a large number of EHPs generated in the i-SiC region by the internal electric field under e-beam irradiation.

**Figure 4:**
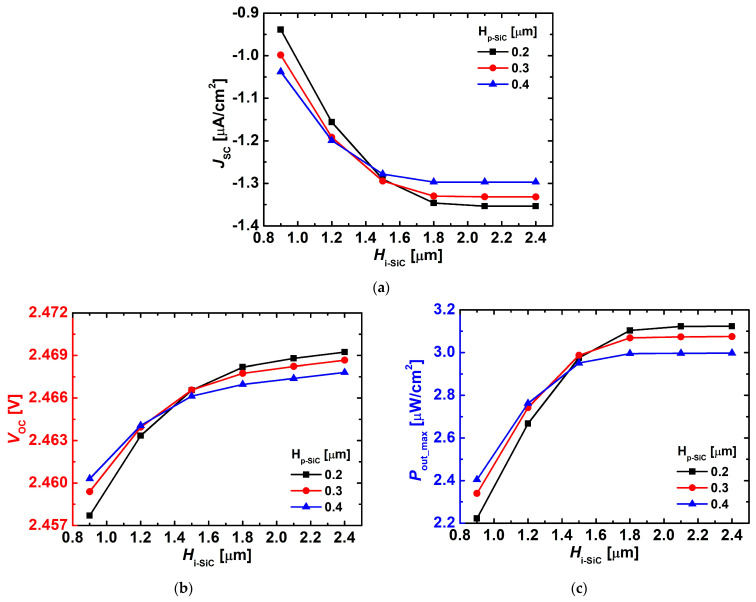
Variations in (**a**) J_SC_, (**b**) V_OC_, and (**c**) P_out_max_ of the simulated 4H-SiC p–i–n diodes as a function of H_i-SiC_ with different H_p-SiC_ (0.2, 0.3, and 0.4 μm). The doping concentrations were D_p-SiC_ = 1 × 10^20^ cm^−3^, D_i-SiC_ = 1 × 10^15^ cm^−3^, and D_n-SiC_ = 1 × 10^20^ cm^−3^.

Figure 4 shows the variations of J_SC_, V_OC_, and P_out_max_ as functions of H_i-SiC_ and H_p-SiC_. As observed in Figure 4a,b, both J_SC_ and V_OC_ gradually increased with increasing H_i-SiC_, since a thicker i-layer allows the generation of more electron–hole pairs under e-beam irradiation. However, the increase of V_OC_ was relatively smaller compared to that of J_SC_. In particular, when H_i-SiC_ exceeded 1.6 μm, the highest J_SC_ and V_OC_ values were obtained under thinner H_p-SiC_ conditions, because a thinner p-layer allows the incident electron beam to reach the i-layer more effectively, resulting in a larger generation of electron–hole pairs.

Figure 4c also reflects this tendency, where P_out_max_ increased with H_i-SiC_ and tended to saturate beyond approximately 2.1 μm. Under these conditions, the highest P_out_max_ of ~3.12 μW/cm^2^ was observed at H_p-SiC_ = 0.2 μm, indicating that an optimized combination of p- and i-layer thicknesses exists for enhancing output power.

**Figure 5:**
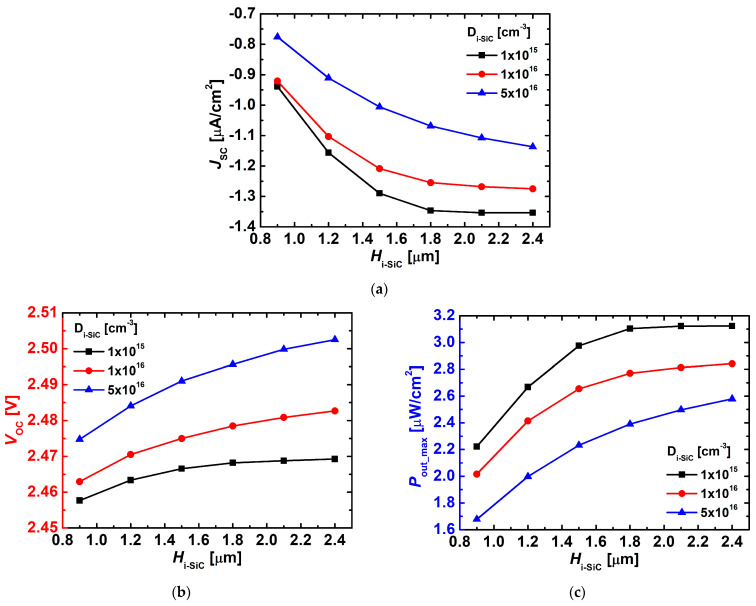
Variations in (**a**) J_SC_, (**b**) V_OC_, and (**c**) P_out_max_ of the simulated 4H-SiC p–i–n diodes as a function of H_i-SiC_ with different D_i-SiC_ (1 × 10^15^, 1 × 10^16^, and 5 × 10^16^ cm^−3^).

Figure 5 presents the variations in J_SC_, V_OC_, and P_out_max_ as functions of H_i-SiC_ and D_i-SiC_. In Figure 5a, J_SC_ increased as D_i-SiC_ decreased, since a lower doping concentration leads to the formation of a wider depletion region, thereby facilitating the separation and transport of electron–hole pairs. Conversely, with higher doping concentrations, the depletion width was reduced and recombination became dominant, resulting in a decrease in J_SC_.

As shown in Figure 5b, V_OC_ increased with higher D_i-SiC_, which can be attributed to the larger built-in potential in the i-layer under higher doping conditions. Although the i-layer is nominally intrinsic, it is lightly doped with n-type impurities. As the i-layer doping concentration (D_i−SiC_) increases, the depletion region becomes narrower and the built-in potential across the i-layer strengthens, thereby leading to a higher open-circuit voltage (V_OC_) despite the fixed dopings of the p- and n-layers [13].

In Figure 5c, P_out_max_ exhibited the highest output power at D_i-SiC_ = 1 × 10^15^ cm^−3^, where the opposing behaviors of J_SC_ and V_OC_ reached a balance.

**Figure 6:**
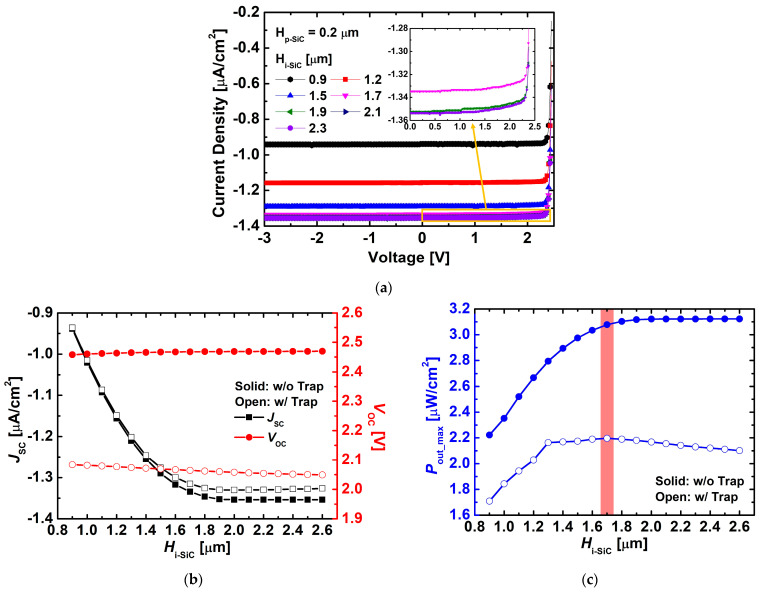
Variations in (**a**) J–V characteristics, (**b**) J_SC_ and V_OC_, and (**c**) P_out_max_ of the simulated 4H-SiC p–i–n diodes as a function of H_i-SiC_, with and without trap states. The trap density was set to 1 × 10^15^ cm^−3^ for both donor- and acceptor-like traps, and the trap energy levels were set to donor-like trap = E_V_ + 1.6 eV and acceptor-like = E_C_ − 0.63 eV.

Figure 6 compares the J–V characteristics, J_SC_, V_OC_, and P_out_max_ as functions of H_i-SiC_, with and without trap states. The trap density was set to 1 × 10^15^ cm^−3^ for both donor- and acceptor-like traps, and the energy levels were defined as donor-like trap = E_V_ + 1.6 eV and acceptor-like trap = E_C_ − 0.63 eV. As shown in Figure 6a, in the absence of traps (w/o trap), the reverse current density gradually increased with increasing H_i-SiC_, since a thicker i-layer facilitates the generation and transport of EHPs. In contrast, Figure 6b,c illustrate detailed variations in J_SC_, V_OC_, and P_out_max_ depending on the presence of traps. In particular, as shown in Figure 6b, J_SC_ exhibited little difference between the trap and no-trap conditions when H_i-SiC_ was thin, but a clear discrepancy emerged beyond ~1.6 μm. This behavior can be attributed to the significantly increased cumulative generation of EHPs in the deeper i-layer under e-beam irradiation, where trap-induced capture and recombination effects become more pronounced in thicker i-layers.

For V_OC_, differences between the trap and no-trap cases were observed across the entire thickness range, with the gap slightly widening as H_i-SiC_ increased. This behavior can be attributed to the reduction in current caused by trap-induced electron-hole recombination, which consequently leads to a decrease in V_OC_. As a result, the presence of traps caused both J_SC_ and V_OC_ to be further degraded.

Regarding P_out_max_, in the absence of traps, saturation occurred when H_i-SiC_ exceeded ~2.0 μm. However, in the presence of traps, the maximum P_out_max_ (~2.20 μW/cm^2^) was obtained at H_i-SiC_ ≈ 1.7 μm, after which device performance deteriorated as the i-layer became thicker. This result suggests that simply increasing the i-layer thickness is not always beneficial, and the actual optimal i-layer thickness should be considered to be ~1.7 μm when trap effects are present. Based on this result, subsequent trap analyses were performed under the conditions of H_p-SiC_ = 0.2 μm, H_i-SiC_ = 1.7 μm, H_n-SiC_ = 2.0 μm, and doping concentrations of D_p-SiC_ = 1 × 10^20^ cm^−3^, D_i-SiC_ = 1 × 10^15^ cm^−3^, and D_n-SiC_ = 1 × 10^20^ cm^−3^.

**Figure 7:**
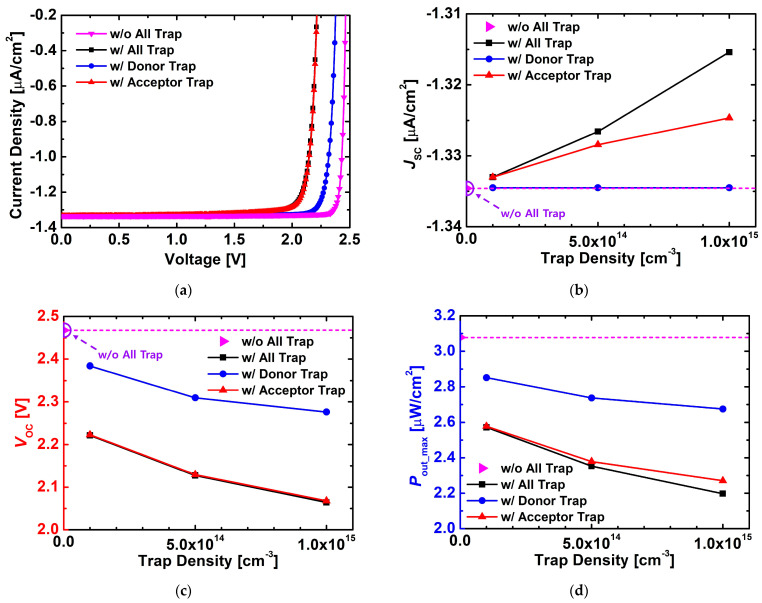
Variations in (**a**) J–V characteristics, (**b**) J_SC_, (**c**) V_OC_, and (**d**) P_out_max_ of the simulated 4H-SiC p–i–n diodes with different trap types (w/o trap, all traps, donor-like trap, and acceptor-like trap) as a function of trap density. The trap density was varied up to 1 × 10^15^ cm^−3^, and the trap energy levels were set to donor-like trap = E_V_ + 1.6 eV and acceptor-like trap = E_C_ − 0.63 eV.

Figure 7 illustrates the changes in the electrical characteristics of the device depending on the trap type (donor or acceptor-like trap), based on the previously optimized structure. As shown in Figure 7a, the device without traps exhibited the most stable current–voltage characteristics, while the presence of donor-like traps resulted in relatively well-maintained current behavior. In contrast, when only acceptor-like traps were present, the current characteristics were significantly degraded, indicating that acceptor-like traps act as major recombination centers.

Figure 7b,c compare the variations in J_SC_ and V_OC_ with increasing trap density. Donor-like traps showed only a minor influence even as their density increased, since they are deep-level traps (E_V_ + 1.6 eV) located near the mid-gap, which exhibit limited recombination activity, particularly under low bias conditions. In contrast, acceptor-like traps (E_C_ − 0.63 eV) caused a gradual reduction in both J_SC_ and V_OC_ their density increased, owing to enhanced carrier recombination near the conduction band. These results confirm that donor traps have a negligible impact on carrier transport, while acceptor traps serve as dominant recombination centers limiting device performance.

As shown in Figure 7d, the results for P_out_max_ followed the same trend. The device without traps exhibited the highest output, and the reduction in output was relatively modest when only donor-like traps were considered. In contrast, acceptor-like traps caused a sharp decrease in output power with increasing trap density. This difference can be explained by the energy levels of the traps: acceptor-like traps (E_C_ − 0.63 eV) are located near the conduction band and thus effectively capture and recombine electrons, whereas donor-like traps (E_V_ + 1.6 eV), positioned near the mid-gap, contribute less to recombination.

Therefore, these results confirm that acceptor-like traps are the dominant cause of performance degradation in actual devices. To realize high-efficiency betavoltaic cells, process control aimed at minimizing the formation of acceptor-like traps is essential.

**Figure 8:**
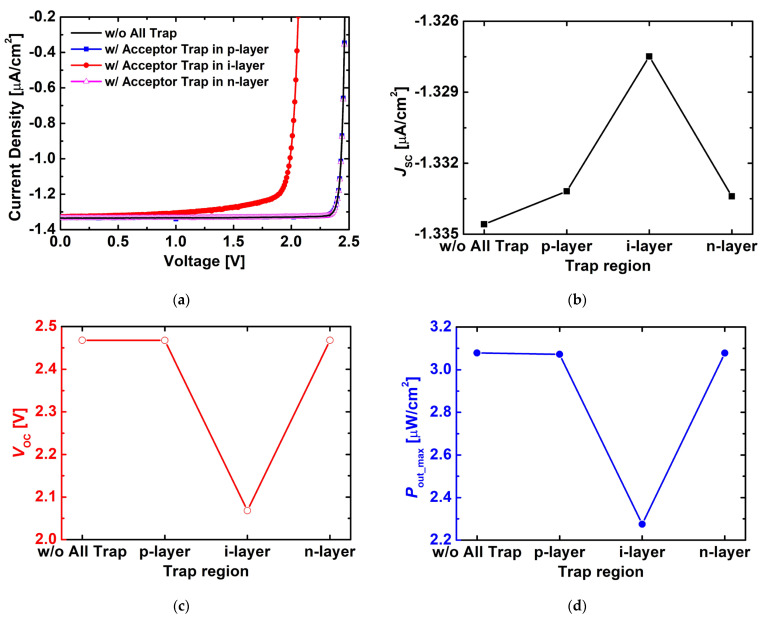
Variations in (**a**) J–V characteristics, (**b**) J_SC_, (**c**) V_OC_, and (**d**) P_out_max_ of the simulated 4H-SiC p–i–n diodes with acceptor traps located in different layers (p-, i-, and n-layer). The trap density was set to 1 × 10^15^ cm^−3^, and the trap energy level was set to acceptor = E_C_ − 0.63 eV.

Figure 8 compares the changes in the electrical characteristics depending on the location of acceptor-like traps (p-, i-, or n-layer). Based on the optimized structure derived earlier, acceptor-like traps (N_t_ = 1 × 10^15^ cm^−3^, E_C_ − 0.63 eV) were individually placed in each layer for analysis. As shown in Figure 8a, when traps were located in the i-layer, both forward and reverse J–V characteristics exhibited the most severe degradation. Figure 8b–d reveal similar trends: J_SC_ decreased most significantly when traps were in the i-layer, while V_OC_ dropped substantially from ~2.47 V to ~2.07 V, and P_out_max_ was clearly reduced from ~3.08 μW/cm^2^ to ~2.28 μW/cm^2^.

In contrast, when traps were located in the p- or n-layer, J_SC_, V_OC_, and P_out_max_ remained comparable to the trap-free case. This is because the i-layer serves as the core region for carrier generation, separation, and transport, while the heavily doped p- and n-type layers contribute negligibly to power generation due to rapid recombination.

Therefore, trap-assisted recombination occurring within the i-layer becomes the dominant mechanism of performance degradation, and controlling acceptor-like traps in this region is essential for achieving high-efficiency betavoltaic cells.

## 4. Conclusions

In this study, structural optimization and trap effect analysis of 4H-SiC p–i–n betavoltaic diodes were systematically performed using TCAD simulations combined with an e-beam irradiation model. Structural optimization was first carried out by varying the i-layer thickness, the p-layer thickness, and the i-layer doping concentration. As a result, the highest P_out_max_ of ~2.20 μW/cm^2^ was obtained at an i-layer thickness of ~1.7 μm, a p-layer thickness of 0.2 μm, and an i-layer doping concentration of 1 × 10^15^ cm^−3^. This condition was then used as the baseline for subsequent trap analyses.

Based on the optimized structure, the effects of trap type and location were systematically examined. The results confirmed that acceptor-like traps act as dominant recombination centers, causing significant reductions in J_SC_, V_OC_, and P_out_max_ compared to donor-like traps. In particular, when acceptor-like traps were located in the i-layer, V_OC_ decreased from ~2.47 V to ~2.07 V, and P_out_max_ dropped from ~3.08 μW/cm^2^ to ~2.28 μW/cm^2^, indicating that the i-layer is the most trap-sensitive region. In contrast, the presence of traps in the p- or n-layer had only a negligible impact on device performance.

These findings demonstrate that the primary cause of performance degradation in practical 4H-SiC betavoltaic cells is the presence of acceptor-like traps in the i-layer. Therefore, minimizing the formation of deep-level acceptor-like traps within the i-layer is essential for realizing high-efficiency and highly reliable betavoltaic cells.

## Figures and Tables

**Table 1:** Representative physical parameters of typical semiconductor materials for BV cell applications [18,19,20,21,22].

Properties	Si	GaAs	GaN	4H-SiC
Bandgap (eV)	1.12	1.42	3.44	3.26
Thermal Conductivity (W/cm·K)	1.45	0.5	2.53	3.7
Breakdown Field (MV/cm)	0.3	0.4	3.3	2.0
Electron Mobility (10^3^ cm^2^/V·s)	1.35	8.5	1.25	0.72
Threshold Displacement Energy (eV)	10–30	13 ± 1 (average)	38 (Ga)/ 16 (N)	38 (Si)/ 19 (C)

## Data Availability

The original contributions presented in this study are included in the article. Further inquiries can be directed to the corresponding authors.
